# Safety and efficacy of cryobiopsy for the diagnosis of lymphangioleiomyomatosis compared with forceps biopsy and surgical lung biopsy

**DOI:** 10.1186/s12890-023-02810-z

**Published:** 2023-12-15

**Authors:** Yao Yao, Xiaobo Chen, Huanjie Chen, Zhulin Xiao, Shiyue Li

**Affiliations:** 1 Department of Pulmonary and Critical Care Medicine, Guangdong Provincial Hospital of Chinese Medicine, Guangdong Provincial Academy of Chinese Medical Sciences, Guangzhou, Guangdong P.R. China 510120; 2 State Key Laboratory of Respiratory Disease, National Clinical Research Center for Respiratory Disease, Guangzhou Institute of Respiratory Health, the First Affiliated Hospital of Guangzhou Medical University, Guangzhou, Guangdong 510120 P.R. China

**Keywords:** Bronchoscopy and interventional techniques, Lymphangioleiomyomatosis, Respiratory function tests, Surgical lung biopsy, Transbronchial lung cryobiopsy, Transbronchial lung forceps biopsy

## Abstract

**Background:**

Transbronchial lung forceps biopsy (TBFB) is recommended before a surgical lung biopsy (SLB) when a definitive diagnosis of lymphangioleiomyomatosis (LAM) is required for patients without any additional confirmatory features. Transbronchial lung cryobiopsy (TBCB) has been suggested as replacement test in patients considered eligible to undergo SLB for the diagnosis of interstitial lung diseases. The efficacy and safety of TBCB were compared with that of TBFB and SLB in the diagnosis of LAM.

**Methods:**

A retrospective analysis was conducted on 207 consecutive patients suspected with LAM in the First Affiliated Hospital of Guangzhou Medical University from 2005 to 2020.

**Results:**

The difference in diagnostic rate of patients suspected with LAM between TBCB (20/30, 66.7%) and TBFB (70/106, 66.0%) groups was not significant (*p* = 0.949). One patient performed TBCB with negative pathological results could be diagnosed exclusively after SLB. LAM diagnosis was confirmed by surgical pathological findings in 3 TBFB-negative patients. More patients with minimal cystic profusion were diagnosed with LAM by TBCB (5/19, 26.3%) and SLB (11/39, 28.2%) than by TBFB (3/61, 4.9%) (TBCB vs TBFB: *p* = 0.04, SLB vs TBFB, *p* < 0.001). The difference between the severity of cystic lung disease in patients diagnosed with LAM through TBCB and SLB was not significant (*p* > 0.05). One pneumothorax, 8 mild bleeding and 1 moderate bleeding were observed in TBCB. One pneumothorax, 15 mild bleeding and 1 moderate bleeding occurred after TBFB.

**Conclusion:**

Compared to TBFB, TBCB is safe and effective in diagnosing LAM at a higher diagnostic rate in patients with minimal cystic profusion.

## Background

Lymphangioleiomyomatosis (LAM), a rare disease occurring in women of reproductive age, is characterized by the presence of multiple, bilateral, uniform, round, and thin-walled cysts present in a diffused distribution, as detected by high-resolution computed tomography (HRCT) of the chest. The estimated prevalence of LAM was between 3.35 and 7.76 cases/million women with an incidence of 0.23–0.31/million women/year [1]. More than 600 cases of LAM had been reported and registered in China by the end of 2017 [2]. Renal angiomyolipoma (AML) is found in 37.8% of patients with LAM, at a rate of 88.2% in tuberous sclerosis complex (TSC) LAM and 29.1% in sporadic LAM [3]. Chylous effusions occurs in 13.8% patients with TSC-LAM and 20% patients with sporadic LAM [4]. Smooth muscle cell infiltration and cystic remodeling of pulmonary parenchyma cause progressive dyspnea, pneumothorax, hypoxemia, and respiratory failure in patients with LAM. Definitive diagnosis is necessary prior to pharmacotherapy. In a woman with a compatible chest CT, a serum VEGF-D concentration of more than 800 pg/mL eliminates the need for an invasive lung biopsy, concentrations between 600 and 800 pg/mL are highly suspicious for LAM, and values of less than 600 pg/mL are considered uninformative and do not exclude LAM [5]. However, commercially available VEGF-D testing remains a global need. If a patient with compatible clinical history and characteristic chest HRCT does not exhibit additional confirmatory features, it necessitates histopathological confirmation of LAM by lung biopsy for definitive diagnosis [5, 6]. Surgical lung biopsy (SLB) has been considered to be the gold standard procedure for obtaining histopathological confirmation of LAM [5, 6]; however, risk associated cannot be ignored. The mortality rate in video-assisted thoracoscopic surgery (VATS)-guided surgical lung biopsy is 1.5 to 4.5%, and procedure-related complication rate is 10 to 19% [7–10]. Compared to SLB, transbronchial lung forceps biopsy (TBFB) is less invasive and a relatively safer procedure with certain false negative rates. Given the relatively lower cost of TBFB, it is recommended prior to SLB [6]. It has been demonstrated that cryobiopsies are considerably larger than forceps biopsies and enable pattern recognition similar to that achieved by SLB [11]. Furthermore, the diagnostic rate for interstitial lung diseases (ILDs) and lung tumors obtained by transbronchial lung cryobiopsies (TBCBs) is higher than that obtained by TBFB [12]. TBCB has been suggested as replacement test in patients considered eligible to undergo SLB with undiagnosed ILD [13]. There are a few cases reports about diagnosis of LAM with TBCB [14, 15]. Research on safety and efficacy of cryobiopsy for the diagnosis of LAM compared with forceps biopsy and surgical lung biopsy is lacking.

We compared the safety and efficacy of TBCB to that of TBFB and SLB in the diagnosis of LAM, and studied the markers of parenchymal LAM burden of patients which may affect the diagnostic rate of LAM at the First Affiliated Hospital of Guangzhou Medical University.

## Methods

### Study population

Medical records of 207 consecutive patients with initial diagnosis of LAM from January 2005 to December 2020 were reviewed from the database of the First Affiliated Hospital of Guangzhou Medical University. The following data were retrieved: age, gender, arterial blood gas analyses in the supine position in room air, number of TBFB/TBCB specimens, pulmonary function test results, and quantitative HRCT images.

A definite diagnosis was confirmed when a patient with compatible clinical history and characteristic chest HRCT had one or more of the following features: presence of TSC, renal angiomyolipoma(s), elevated serum VEGF-D ≥ 800 pg/ml, chylous effusion (pleural or ascites) confirmed by tap and biochemical analysis of the fluid, lymphangioleiomyomas, presence of LAM cells or LAM cell clusters in cytological examination of effusions or lymph nodes, and histopathological confirmation of LAM by lung biopsy or biopsy of retroperitoneal or pelvic masses [6].

This study was approved by the Ethics Committee of the First Affiliated Hospital of Guangzhou Medical University.

### Cryobiopsy and forceps biopsy

Since TBCB was conducted in our hospital, TBCB or TBFB were performed on patients as per their choice. Physicians recommended TBCB over TBFB.

TBCB: Flexible bronchoscopy was performed via rigid bronchoscopy using a flexible 1.9 mm cryobiopsy probe (Erbe, Tübingen, Germany) under general anesthesia. Freezing time was 4–6 seconds in order to achieve the same specimen size. Biopsies were taken in the most affected areas [16]. The specimen was extracted along with the bronchoscope for thawing and subsequent processing. Physicians attempted to collect 5 specimens from each patient.

TBFB: TBFB was performed using an Olympus BF-260 on patients under conscious sedation by administration of sufentanil and midazolam. Physicians attempted to collect 5 specimens from each patient.

TBCB and TBFB were conducted by one of the 3 physicians in the department of bronchoscopy randomly. Each physician has over 3 years of total experience performing over 100 TBCBs and over 5 years of cumulative experience performing over 2000 TBFBs.

No bleeding was defined as the presence of only traces of blood after finishing the biopsies, with no need for continued suctioning. Mild bleeding was defined as the need for continued suctioning of blood from the airways after the procedure, and moderate bleeding required intubation of the biopsied segment with the flexible bronchoscope into the wedge position. Severe bleeding was defined as the need for additional interventions, such as placing a temporary bronchus blocker, applying a fibrin sealant, admission to a critical care unit, or infusing blood products [17].

### HRCT

The lung LAM disease was graded on the basis of severity of cystic lung disease (Grade I: minimal, less than 30% abnormal; Grade II: moderate, 30–60% abnormal; and Grade III: severe, more than 60% abnormal) by three radiologists who were unaware of final diagnosis [18, 19]. Final grade was determined by the majority.

### Pulmonary function test

All pulmonary function tests were performed using a JAEGER MasterScreen (German) or Cosmed Quark PFT (Italia). Diffusing capacity of carbon monoxide (DLCO) was measured using the single-breath method with correction for hemoglobin; total lung capacity (TLC) and residual volume (RV) were determined using plethysmography.

### Pathology

Biopsy specimens were reviewed by experienced pathologists in LAM and stained for markers including smooth muscle actin (SMA), estrogen receptor (ER) and progesterone receptor (PR) and HMB-45.

### Statistical analysis

All data were analyzed using statistical software SPSS version 22.0 (SPSS Inc., Chicago, IL, USA). The distribution of variables was assessed by means of a Kolmogorov-Smirnov goodness-of-fit test. Groups were compared by one-way ANOVA with Bonferroni correction for multiple comparisons or by the Kruskal-Wallis H test with post hoc tests applying the Nemenyi test for multiple comparisons. Categorical variables were compared using the Chi-squared test or Fisher’s exact test. *P* < 0.05 was considered statistically significant.

## Results

### Patient demographics and pulmonary function

All 207 patients suspected with LAM were female and the median age was 40(range 18–67). Eleven patients (5.3%) were TSC-LAM. There were 77 patients (37.2%) with history of pneumothorax. Ten patients (4.8%) had pleural chylous effusion. Patients underwent SLB during surgical treatment of pulmonary bullae or spontaneous pneumothorax with or without VATS. The rate of patients with past history of pneumothorax was higher in patients diagnosed LAM by SLB (hereafter referred to as SLB-positive patients) than in patients diagnosed LAM by TBCB (hereafter referred to as TBCB-positive patients) and TBFB (hereafter referred to as TBFB-positive patients) (*p* < 0.017) (Table 1). Fewer specimens were collected from TBCB-positive patients than from patients diagnosed LAM by TBFB (*p* = 0.016) (Table 1). There were no significant differences in age, SpO2 and smoking history among TBFB-positive, TBCB-positive, and SLB-positive patients (Table 1). TBFB-positive patients had lower FEV1/FVC of compared with SLB-positive patients(*p* = 0.03) (Table 1). The %predicted TLC in SLB-positive patients was lower than that in TBFB-positive and TBCB-positive patients (*p* < 0.05) (Table 1). There was no significant difference in %predicted DLco, %predicted FEV1, %predicted RV, and RV/TLC among TBFB-positive, TBCB-positive, and SLB-positive patients (Table 1).

**Table 1:** Patient demographics and pulmonary function of patients

	TBFB+ (*n* = 70) count (%) median (range)	TBCB+ (*n* = 20) count (%) median (range)	SLB+ (*n* = 45) count (%) median (range)	*p* value			
				All	TBFB+ vs. TBCB+	TBFB+ vs. SLB+	TBCB+ vs. SLB+
Gender							
Female	70 (100%)	20 (100%)	45 (100%)				
Age	38 (20,60)	36 (25,54)	36 (18,61)	0.713			
PaO_2_(Torr)	96 (84,99) (*n* = 56)	97 (86,99) (*n* = 20)	97 (88,100) (*n* = 35)	0.312			
Smoking history				0.800			
Never	68 (97.1%)	20 (100%)	42 (97.7%)				
Current or ex-smoker	2 (2.9%)	0 (0%)	1 (2.3%)				
Past history of pneumothorax				0.001	0.123	**0.003**	**< 0.001**
Yes	27 (38.6%)	4 (20.0%)	29 (67.4%)				
No	43 (61.4%)	16 (80.0%)	14 (32.6%)				
Chylous effusions	4 (5.7%)	0 (0.0%)	4 (5.7%)	0.202			
Tuberous sclerosis complex	3 (4.3%)	2 (10.0%)	2 (4.7%)	0.639			
Renal angiomyolipoma	10 (14.3%)	7 (35.0%)	6 (14.0%)	0.109			
Number of specimens	5.5 (4,10) *n* = 20	4 (3,5) *n* = 12			**0.016**		
%predicted DLco	42.50 (18.00,119.00) *n* = 54	50.00 (24.00.94.80) *n* = 19	56.00 (12.00,97.00) *n* = 25	0.384			
%predicted FEV_1_	58.00 (14.50,105.10) *n* = 55	77.00 (23.60,138.70) *n* = 20	62.00 (18.00,99.60) *n* = 31	0.259			
FEV_1_/FVC	58.52 (21.03,99.61) *n* = 55	70.40 (22.74,87.10) *n* = 20	76.62 (36.80,98.68) *n* = 31	0.030	0.740	**0.030**	0.360
%predicted TLC	101.50 (64.00,138.90) *n* = 50	105.00 (84.00,142.50) *n* = 19	89.15 (59.10,124.80) *n* = 22	0.013	0.999	**0.036**	**0.020**
%predicted RV	125.05 (67.00,326.20) *n* = 50	127.00 (58.00,257.80) *n* = 19	117.00 (74.00,254.00) *n* = 22	0.736			
RV/TLC	39.60 (22.58,77.18) *n* = 50	38.80 (17.50,65.57) *n* = 19	39.95 (28.80,68.88) *n* = 22	0.634			

*TBFB* transbronchial lung forceps biopsy, *TBCB* transbronchial lung cryobiopsy, *SLB* surgical lung biopsy, *+* positive, − negative, *PaO*_*2*_ partial pressure of arterial oxygen, *DL*_*CO*_ diffusing capacity of carbon monoxide, *FEV*_*1*_ forced expiratory volume in 1 s, *FVC* forced vital capacity, *RV* residual volume, *TLC* total lung capacity

### Diagnosis of LAM

**Fig. 1:**
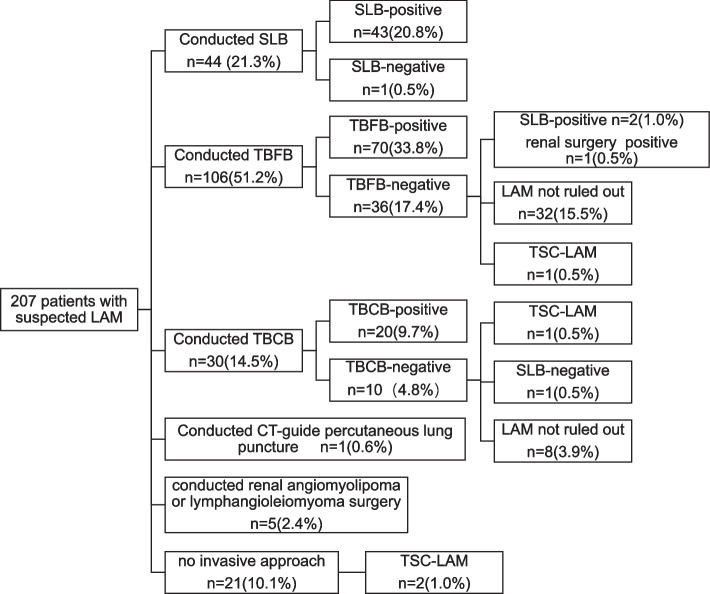
Diagnostic process for LAM; LAM, lymphangioleiomyomatosis; SLB, surgical lung biopsy; TBFB, transbronchial lung forceps biopsy; TBCB, transbronchial lung cryobiopsy

Diagnosis of LAM was confirmed in 146 (70.5%) patients. Twenty-one (10.1%) patients refused any invasive approaches, two of which were TSC-LAM. Sixty-one (29.5%) patients were suspected to be suffering from LAM at the time of discharge. TBCB was conducted on 30 patients, of which 20 patients (66.7%) demonstrated characteristic pathological findings of LAM. The diagnosis of LAM was excluded by SLB in 1 TBCB-negative patient. TBFB was conducted in 106 patients, of which 70 patients (66.0%) demonstrated characteristic pathological findings of LAM. The diagnosis of LAM was confirmed by surgical pathological findings in 3 TBFB-negative patients (2.9%). Two patients underwent TBFB twice; however, negative pathological results did not rule out the diagnosis of LAM at discharge. Specimen deficiency occurred in 2 cases of TBFB. The difference in diagnostic rate of patients suspected with LAM between TBCB and TBFB groups was not significant (*p* = 0.949). Forty-five patients were diagnosed with LAM after thoracic surgery, of which 26 patients underwent thoracic surgery at other facilities. However, LAM was diagnosed at our respiratory pathology center using borrowed specimens (Fig. 1).

### Chest CT

**Fig. 2:**
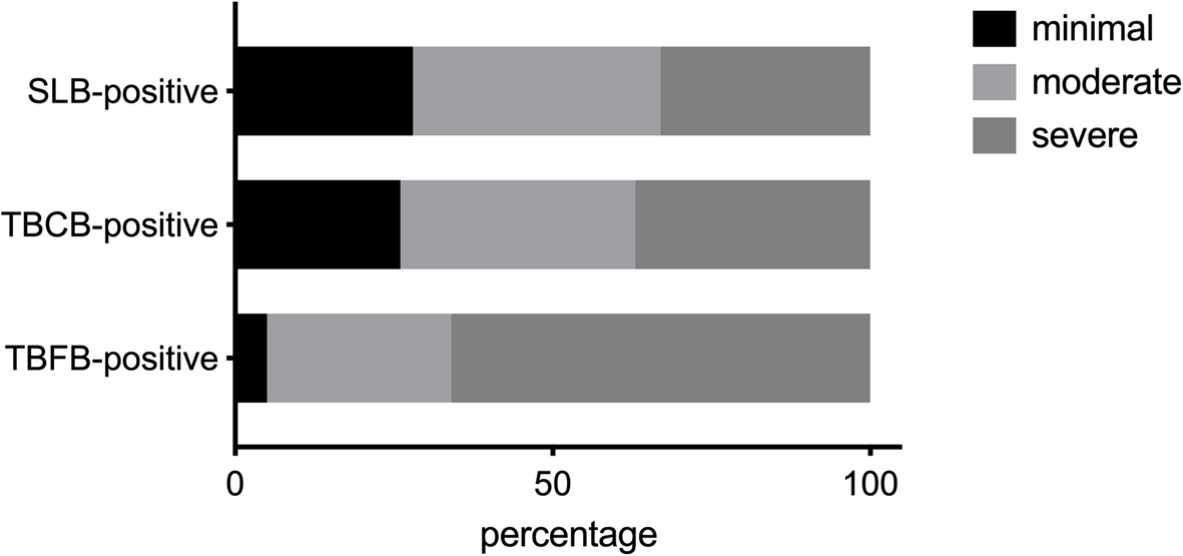
Chest CT manifestation of different severity of cystic lung disease of TBFB-positive, TBCB-positive, and SLB-positive patients graded by radiologist; LAM, lymphangioleiomyomatosis; SLB, surgical lung biopsy; TBFB, transbronchial lung forceps biopsy; TBCB, transbronchial lung cryobiopsy

A greater number of patients with minimal cystic profusion graded by the radiologists were diagnosed with LAM by TBCB (5/19, 26.3%) and SLB (11/39, 28.2%) than by TBFB (3/61, 4.9%). A greater number of patients with severe cystic profusion were diagnosed with LAM by TBFB (40/61, 65.6%) than by SLB (13/39, 33.3%) and TBCB (7/19, 36.8%). (TBCB vs TBFB: *p* = 0.040, SLB vs TBFB, *p* < 0.001) (Fig. 2). The difference between the severity of cystic lung disease in patients diagnosed with LAM through TBCB and SLB was not significant (*p* = 0.97) (Fig. 2). In 22 patients with minimal cystic lung disease, 5 patients (22.7%) chose TBCB, 4 patients (18.2%) chose TBFB and 11 patients (50.0%) chose SLB. The diagnosis of LAM was confirmed by surgical pathological findings in 1 TBFB-negative patients with minimal cystic lung disease. In 41 patients with moderate cystic lung disease, 7 patients (17.1%) chose TBCB, 18 patients (43.9%) chose TBFB and 7patients (17.1%) chose SLB. In 61 patients with severe cystic lung disease, 7 patients (11.5%) chose TBCB, 40 patients (65.6%) chose TBFB and 13 patients (21.3%) chose SLB.

### Safety

One patient (1/30,3.3%) experienced pneumothorax after TBCB. The cystic profusion of this patient was graded as severe by the radiologist. Eight patients (8/30,26.7%) experienced mild bleeding and 1 patient (1/30,3.3%) experienced moderate bleeding after TBCB. No other adverse events, such as life-threatening bleeding after TBCB, were recorded. One pneumothorax (1/106,0.9%),15 mild bleeding (15/106,14.1%) and 1 moderate bleeding (1/106,0.9%) occurred after TBFB. The cystic profusion of the patient with pneumothorax after TBFB was graded as minimal by the radiologist. Biopsy-associated bleeding did not require any intervention. Pneumothorax occurred in 1 patient (1/44,2.3%) with moderate cystic profusion after SLB. No serious adverse events associated with surgery happened in this study population. All pneumothoraxes were resolved by performing tube thoracostomy and complete recovery within a week was ensured.

## Discussion

This study firstly reports the safety and efficacy of TBCB, compared to that of TBFB and SLB, in the diagnosis of LAM, it was discovered that a greater number of patients with minimal cystic profusion were diagnosed with LAM using TBCB and SLB, than by using TBFB.

Gupta et al. reported a diagnosis of LAM by TBCB [14]. Yoshida et al. reported 2 cases of LAM with relatively early-stage cystic lung lesions, diagnosed pathologically using TBCB [15]. Studies on the safety and diagnostic yield of TBCB for patients suspected with LAM are currently lacking. In this study, the difference between diagnostic rate of patients with suspected LAM was not significant, however fewer specimens were collected from TBCB-positive patients than from TBFB-positive patients. Specimen deficiency occurred in TBFB, which might have led to negative pathological results. Multiple studies reported that compared to TBFBs, TBCBs were larger in size, with fewer artifacts and with more alveolar parts, leading to a higher diagnostic rate in interstitial lung disease (ILD), lung tumor, and lung allograft [20–29].

Apart from morphological and histochemical difference between TBCB and TBFB, parenchymal LAM burden of patients may also affect the diagnostic rate. In this study, severity of cystic lung disease manifesting on Chest CT as one of the markers of parenchymal LAM burden was analyzed. Gradation of lung LAM disease was based on severity of cystic lung disease by radiologists who was unaware of the final diagnosis [18, 19]. A greater number of patients with minimal cystic profusion were diagnosed with LAM by applying TBCB and SLB rather than by applying TBFB. The results indicate that to obtain a higher rate of positive pathological results of LAM, TBCB should be performed on patients with minimal cystic profusion manifestation on chest CT. Most of the patients with severe cystic lung disease chose TBFB. Patients tended to choose less invasive procedure. Therefore, more patients with severe cystic profusion were diagnosed with LAM by TBFB. While most patients with severe cystic profusion were diagnosed by TBFB, patients diagnosed by TBFB demonstrated the lowest SpO_2_.

Lung function is another marker of parenchymal LAM burden. Most patients diagnosed by SLB took lung function tests after the surgery, which might indicate the reason behind the decreased %predicted TLC. Koba et al. found that TBLB-positive LAM patients had lower % predicted DLCO than TBLB-negative LAM patients [30]. However, we did not find significant difference in percentage predicted DLCO or in any other pulmonary function parameters between TBFB-positive, TBCB-positive, and SLB-positive patients. TBFB-negative and TBCB-negative LAM patients should be included in future research. Airflow obstruction is the most common physiological manifestation of LAM. However, we found that TBFB-positive patients had lower FEV1/FVC which occurred in late stages of disease compared with SLB-positive patients suggesting that TBFB had a low diagnostic yield in early-stage cases.

To date, none of the studies have compared the diagnostic value of TBCB in LAM with that of SLB. The former comparisons between TBCB and SLB were made in ILD. Hagmeyer et al. determined that conducting SLB was unnecessary after TBCB in 38/51 patients (75%), and in 12/13 patients (92%); SLB conformed results obtained from TBCB [31]. Ravaglia et al. performed a retrospective study on 447 patients with ILD and determined that the diagnostic rate of TBCB and SLB to be 82.8 and 98.7% respectively, with a significant difference [32]. Tomassetti et al. conducted a cross-sectional study and detected an increase in diagnostic confidence following a comparable performance of TBCB and SLB and a similar interobserver agreement in diagnosis of idiopathic pulmonary fibrosis (IPF) diagnosis [33]. Romagnoli et al. conducted a two-center prospective study in which patients with ILD underwent both TBCB and SLB, and found poor concordance between TBCB and SLB in the assessment of ILD, with SLB demonstrating higher frequency of concordance with the final diagnosis [34]. Rodrigues et al. conducted systematic review and meta-analysis of 43 studies and concluded that TBCB should be considered as an alternative to SLB or at least as a first-line procedure for lung tissue sampling [35]. In this study, we firstly compared efficacy of TBCB and SLB in LAM diagnosis and discovered that the cystic profusion severity of patients diagnosed with LAM was similar using both TBCB and SLB, signifying that TBCB may dispense with the need of SLB in some patients with minimal parenchymal LAM burden. Patients undergoing both TBCB and SLB should be studied before TBCB can be considered as a substitute for SLB in LAM.

Pneumothorax and bleeding are major complications after TBCB or TBFB. Koba et al. and Torre et al. reported no occurrence of pneumothorax after TBFB in 7 and 24 consecutive LAM patients, respectively [30, 36]. In the present study, one pneumothorax occurred after TBFB in a patient with moderate cystic parenchymal burden. One patient with severe cystic profusion, as graded by the radiologist, experienced pneumothorax after TBCB. The rate of pneumothorax occurrence may decrease in patients with severe cystic profusion if TBCB is avoided. No fatal bleeding occurred after TBFBs or TBCBs. Gershman et al. analyzed 402 biopsy procedures in lung allograft recipients and concluded that the rate of pneumothorax and bleeding after TBCB and TBFB were comparable [37]. Pajares et al. found that in ILD, grade 2 bleeding was more frequent (not statistically significant) in the TBCB group (56.4%) than in the TBFB group (34.2%), but no differences were observed in the frequency of other complications [26]. LAM varies significantly from ILD or lung allograft as one of cystic lung diseases. Nevertheless, rate of complications after TBFB and TBCB need to be compared in a greater number of LAM patients, as different parenchymal LAM burdens act as an important risk factor of pneumothorax. Ravaglia et al. performed a retrospective analysis on 447 patients with ILD, and observed mortality due to adverse events in 2.7% (SLB) and 0.3% (TBCB) of the patients [32]. Rodrigues et al. found that the mortality rate of patients with ILD reported in 29 studies was 0.6% (range 0–3.2%) and 1.7% (range 0–6.7%) in TBCB and SLB, respectively [35]. Compared to SLB, TBCB is safe and with lower complication and mortality rates. However, there were no significantly adverse events after surgery in this study population. A major number of patients underwent thoracic surgery at a different facility; hence, details of any other adverse events were not available.

Some limitations should be taken into consideration. First, it was a retrospective study, we did not analyze serum VEGF-D concentration, specimen size. Second, we had no comparisons of TBCB with TBFB in same patient. Furthermore, a prospective, randomized clinical trial is needed in the further study.

## Conclusion

Overall, compared with TBFB, a greater number of LAM patients with minimal cystic profusion were diagnosed by TBCB. We suggested that patients with minimal parenchymal cysts burden Chest CT manifestation better be performed TBCB for higher diagnostic rate of LAM. TBCB may dispense with the need for SLB in some patients with minimal parenchymal LAM burden.

## Data Availability

The datasets used and/or analysed during the current study are available from the corresponding author on reasonable request.

## Abbreviations

TBFB: transbronchial lung forceps biopsy
SLB: surgical lung biopsy
LAM: lymphangioleiomyomatosis
TBCB: transbronchial lung cryobiopsy
HRCT: high-resolution computed tomography
VATS: video-assisted thoracoscopic surgery
ILDs: interstitial lung diseases
TSC: tuberous sclerosis complex
DLCO: diffusing capacity of carbon monoxide
TLC: total lung capacity
RV: residual volume
PaO2: partial pressure of arterial oxygen
FEV1: forced expiratory volume in 1 s
FVC: forced vital capacity
RV: residual volume.
